# Characterization of a new model of chemotherapy-induced heart failure with reduced ejection fraction and nephrotic syndrome in Ren-2 transgenic rats

**DOI:** 10.1038/s41440-024-01865-7

**Published:** 2024-09-09

**Authors:** Olga Gawrys, Šárka Jíchová, Matúš Miklovič, Zuzana Husková, Soňa Kikerlová, Janusz Sadowski, Petra Kollárová, Olga Lenčová-Popelova, Lenka Hošková, John D. Imig, Yvona Mazurova, František Kolář, Vojtěch Melenovský, Martin Štěrba, Luděk Červenka

**Affiliations:** 1 Center for Experimental Medicine, Institute for Clinical and Experimental Medicine, Prague, Czech Republic; 2 Department of Pharmacology, Faculty of Medicine in Hradec Králové, Charles University, Hradec Králové, Czech Republic; 3 Department of Cardiology, Institute for Clinical and Experimental Medicine, Prague, Czech Republic; 4 Department of Pharmaceutical Sciences, College of Pharmacy, University of Arkansas for Medical Sciences, Little Rock, AR USA; 5 Department of Histology and Embryology, Faculty of Medicine in Hradec Králové, Hradec Králové, Czech Republic; 6 Laboratory of Developmental Cardiology, Institute of Physiology of the Czech Academy of Sciences, Prague, Czech Republic; 7 Department of Internal Medicine I, Cardiology, University Hospital Olomouc and Palacký University, Olomouc, Czech Republic

**Keywords:** Doxorubicin, Chemotherapy induced heart failure, Ren-2 transgenic hypertensive rat, Experimental model of heart failure, NO/sGC/cGMP pathway

## Abstract

All anthracyclines, including doxorubicin (DOXO), the most common and still indispensable drug, exhibit cardiotoxicity with inherent risk of irreversible cardiomyopathy leading to heart failure with reduced ejection fraction (HFrEF). Current pharmacological strategies are clearly less effective for this type of HFrEF, hence an urgent need for new therapeutic approaches. The prerequisite for success is thorough understanding of pathophysiology of this HFrEF form, which requires an appropriate animal model of the disease. The aim of this study was to comprehensively characterise a novel model of HF with cardiorenal syndrome, *i.e*. DOXO-induced HFrEF with nephrotic syndrome, in which DOXO was administered to Ren-2 transgenic rats (TGR) *via* five intravenous injections in a cumulative dose of 10 mg/kg of body weight (BW). Our analysis included survival, echocardiography, as well as histological examination of the heart and kidneys, blood pressure, but also a broad spectrum of biomarkers to evaluate cardiac remodelling, fibrosis, apoptosis, oxidative stress and more. We have shown that the new model adequately mimics the cardiac remodelling described as “eccentric chamber atrophy” and myocardial damage typical for DOXO-related cardiotoxicity, without major damage of the peritoneum, lungs and liver. This pattern corresponds well to a clinical situation of cancer patients receiving anthracyclines, where HF develops with some delay after the anticancer therapy. Therefore, this study may serve as a comprehensive reference for all types of research on DOXO-related cardiotoxicity, proving especially useful in the search for new therapeutic strategies.

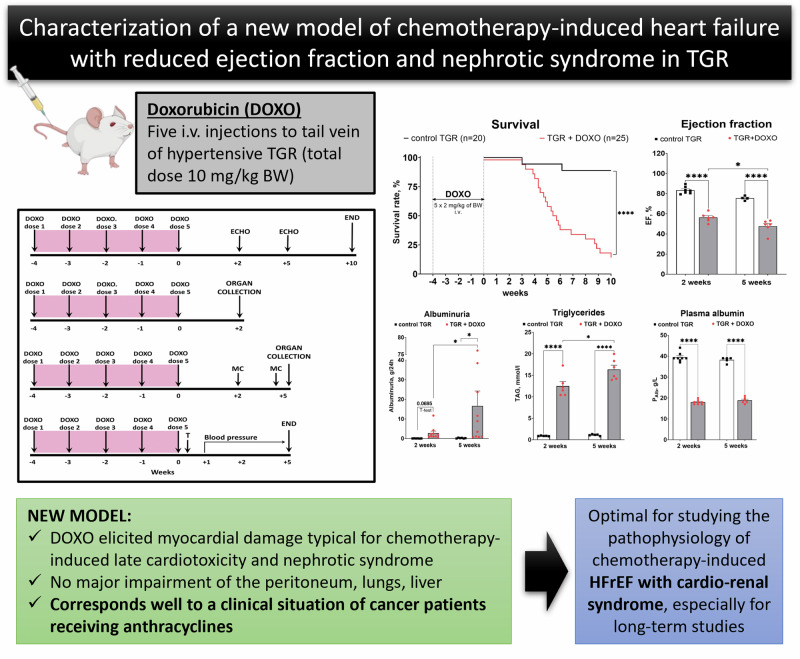

## Introduction

Advances in cancer therapy have markedly improved treatment outcomes, particularly increasing the long-term survival. However, chronic toxicities of cancer therapies substantially reduce the quality of life and sometimes also the patients’ life expectancy [1]. This is the case of anthracyclines: they brought a major breakthrough in oncology and are a cornerstone of treatment of a variety of cancers, including several paediatric malignancies [1–3]. However, doxorubicin (DOXO), the most common anthracycline used in the clinic, is known to induce cardiotoxicity with an inherent risk of largely irreversible cardiomyopathy, leading to reduction of left ventricular (LV) ejection fraction (LVEF) and heart failure (HFrEF) [1, 4–6]. In addition, DOXO was reported to cause nephrotoxicity, both in cancer patients and in experimental animals [7–9]. In the year 2020 the number of childhood cancer survivors treated with anthracyclines was 500 000 in the United States and 650 000 in the European Union [3, 5, 10]. Despite the efforts for refinement of the therapeutic regimens [reduction of DOXO dose, slowed-down infusion, special formulations (liposomal DOXO), or administration of cardio-protective drugs], the risk of DOXO-caused cardiotoxicity is still serious and may be further complicated by kidney damage [1, 2, 11].

DOXO-induced toxicity considerably increases the risk of premature cardiovascular disease [1, 10, 12], with 15fold higher risk of HFrEF than in siblings without anthracycline treatment [1, 13]. Childhood cancer survivors display particularly high risk for specific health-related causes of death [14]. At least 10% of long-term childhood cancer survivors can be endangered by the development of “chemotherapy-induced HFrEF” at the age of approximately 40 years [1, 3]. In addition, nephrotoxicity is one of the escalating late-term side effects of all anticancer therapies, including DOXO [11]. The signs of the resultant acute or chronic kidney disease include tubulopathy, thrombotic microangiopathy (TMA) and may lead to nephrotic syndrome [8, 15].

The guideline-directed medical therapy (GDMT) for HFrEF is now well established and reasonably successful [16–18]. However, currently available pharmacological strategies appear inadequate for effective management of “chemotherapy-induced HFrEF”, especially when it is accompanied by impairment of renal function (cardiorenal syndrome), where dysfunction of one organ may induce or worsen the condition of the other [19]. Therefore, there is an urgent need for new pharmacological therapies to address chemotherapy-induced HFrEF with cardiorenal syndrome. This requires thorough understanding of the pathophysiology of this specific HFrEF form based on application of translational preclinical animal models [20, 21]. However, the in vivo modelling of chemotherapy-induced HFrEF is still inadequately standardized and settled [22–24]. Previous relevant studies often used short-term animal models of acute and subacute anthracycline cardiotoxicity and little attention was paid to late consequences of chronic DOXO cardiotoxicity, which is the most important problem with chemotherapy-induced HFrEF in clinical practice [22–27].

In this study we employed hypertensive Ren-2 transgenic rats (TGR), which bear the additional murine Ren-2 gene cloned from the DBA/2 J mouse strain and they constitute a monogenic model of hypertension of known origin [28]. Due to renin-angiotensin-aldosterone overactivation and marked hypertension, which are the most common comorbidities in both cancer and HF patients, TGR rats are suspected to be more sensitive towards chemotherapy-induced HFrEF and renal failure development. This might enhance studying later consequences of chemotherapy-induced HF in a relatively shorter timeline and without requirement of high cumulative doses which often brings the limitations associated with high nonspecific general toxicity burden [29–34]. In our earlier studies [35, 36] the cumulative intraperitoneal dose of 15 mg/kg of BW was adopted from literature [37, 38]. The limitations of this approach included signs of the peritoneum injury, fibrosis and severe liver damage. Such HFrEF-unrelated morbidity and mortality in this model makes it unsuited for long-term follow up. Therefore, in the present study we employed intravenous DOXO administration once a week, to be closer to situation in clinical practice and also in the translationally oriented experimental in vivo studies [25, 39, 40].

Our aim was to define the pathophysiological details of this DOXO-induced model of HFrEF with cardiorenal syndrome in TGR, with the focus on the very early (2 weeks), early (5 weeks) and the late (survival experiments up to 10 weeks) phase after termination of DOXO administrations.

## Methods

### Ethical approval, animals

The studies were performed in accordance with the guidelines and practices established by the *Animal Care and Use Committee of the Institute for Clinical and Experimental Medicine* (IKEM, Prague), which accord with the ARRIVE guidelines and were carried out in accordance with the EU Directive 2010/63/EU for animal experiments *European Convention on Animal Protection and Guidelines on Research Animal Use*, and finally approved by the Ministry of Health of the Czech Republic (project decision 36388/2019-4/). Studies were performed in male heterozygous TGR that were generated by breeding male homozygous TGR with female homozygous transgene-negative Hannover-Sprague Dawley rats. Previous research show that male rats are far more sensitive to doxorubicin than females [41], hence for this first characterisation study we have selected only male animals, however we are aware that studies in both sexes should be performed in the future. Rats at the initial age of 6 weeks were randomly assigned to experimental groups to make sure that the animals from a single litter do not prevail in any group.

### Preparation of the model of chemotherapy-induced heart failure with reduced ejection fraction (HFrEF) and cardiorenal syndrome: doxorubicin (DOXO) injections

**Fig. 1:**
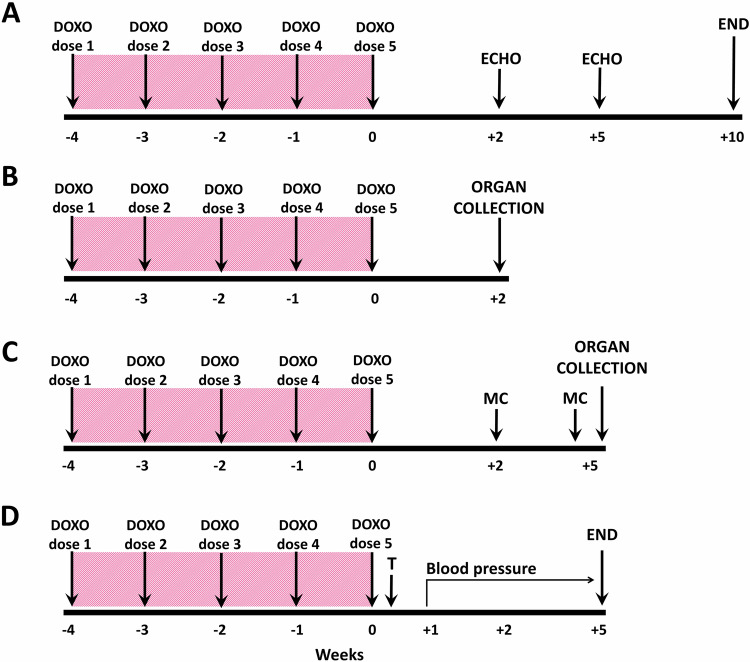
The experimental design. DOXO doxorubicin, ECHO echocardiography, MC metabolic cage with urine collection, T telemetry probes implantation; DOXO was administered intravenously to the tail vein under isoflurane anaesthesia (cumulative dose 10 mg/kg of BW; one injection per week for five consecutive weeks)

TGR rats at the initial age of 6 weeks were anaesthetized with 4% isoflurane (AErrane 100% Liquid Inhalation Vapour, Baxter S.S.Bd.R. Belgium) in the induction phase and maintained by mask inhalation at 1.5–2.0% during the procedure. Oxygen flow equalled 1 l/min; if necessary, the dosage was slightly adjusted, depending on the animal´s weight, its reaction and breathing. The treatment was delivered using Harvard Apparatus Isoflurane Funnel-Fill Vaporizer, Harvard Apparatus, Massachusetts, United States. The rats were placed on the heating pad (37 ^o^C). 1 ml of doxorubicin solution containing a dose of 2 mg/kg of body weight (BW) was prepared from stock solution (2 mg/ml Teva Pharmaceuticals CR, s.r.o. Prague, Czech Republic) in sterile saline and slowly injected into the tail vein through *i.v*. cannula (BD Neoflon^TM^, 24 GA, 0.7 × 19 mm, Becton Dickinson Czechia, s.r.o.). The procedure was repeated weekly for five consecutive weeks to obtain a cumulative dose of 10 mg/kg of BW. Control animals received 1 ml of saline each week. The preparation of the rats with DOXO-induced HFrEF was the same for each experimental series described below and it is depicted in (Fig. 1A–D).

### Series 1—The course of survival and echocardiographic assessment of cardiac function in DOXO-induced model of HFrEF and cardiorenal syndrome

The whole experimental design for the present series is depicted in Fig. 1A. After the last DOXO injection (week labelled 0) the observation started, and the follow-up period was 10 weeks. BW was monitored on the daily basis. Two and five weeks after the last injection of DOXO (weeks labelled +2 and +5) echocardiographic examination was performed and assessed as described in detail in our previous publications [36, 42, 43]. Briefly, the animals were anesthetized with isoflurane (as described above) and the ventral thorax was shaved. For standard measurements of cardiac parameters, B-MODE and M-Mode images were recorded in parasternal long axis and parasternal short axis view at the papillary muscle level. Morphological parameters of the LV and right ventricle (RV), including dimension of LV inner diameter, anterior and posterior walls were measured in M-mode from long and short axis sections as previously described [36, 42, 43]. All ultrasound studies were done by Vivid System (probe 10S, 5–11.5 MHz, Vivid 7 Dimension, GE Healthcare, Chicago, USA) and analysed in EchoPAC software (version 110.1.2 GE Healthcare, Chicago, USA). For each parameter the mean of three optimally obtained measurements in each rat was used. The following experimental groups of animals were examined:

1. control TGR (*n* = 20)
2. TGR + DOXO (*n* = 25)

### Series 2 and 3—Evaluation of the effects of DOXO administration on cardiac damage, liver and renal function

The experimental design for the present series is given in Fig. 1B, C. The same experimental groups (*n* = 5–8 in each) as described in series 1 were exposed to the same protocol of DOXO administration. In Series 2 (Fig. 1B) the rats were killed by decapitation two weeks after the last injection of DOXO (week labelled +2), and in Series 3 after five weeks (Fig. 1C). Additionally, in Series 3 rats were placed twice in metabolic cages for 24 h urine collection (during weeks +2 and +5 before decapitation). The time points for urine collection and decapitation in Series 2 and 3 were selected based on the results of Series 1 where the survival was evaluated. As we saw that the animals started to die already four weeks after the last administration of DOXO, it was important to characterize the changes in the very early phase (week +2) and the early phase (week +5) of the heart failure development.

In both Series 2 and 3 the animals were killed by decapitation and blood samples were collected to measure haematocrit and a spectrum of biomarkers of the kidney (plasma creatinine), liver (plasma albumin) and heart (cardiac troponin T) injury and function. The organs (the liver, heart, kidneys, lungs) were weighed and tissue samples (heart and kidneys) were collected for further analysis to evaluate the state of fibrosis, inflammation, oxidative stress and the degree of activation of neurohormonal regulatory systems: sympathetic nervous system (SNS) and the nitric oxide (NO)/soluble guanylyl cyclase (sGC)/cyclic guanosine monophosphate (cGMP) pathway.

### Series 4—Evaluation of the effects of DOXO administration on blood pressure

The experimental design for Series 4 is given in Fig. 1D. The animals were treated by DOXO or saline as in previous experiments (*n* = 5 in each) and one week after the last DOXO administration telemetry probes were implanted into femoral artery under intraperitoneal ketamine/midazolam anaesthesia Calypsol, Gedeon Richter, Hungary, 160 mg/kg and Dormicum, Roche, France, 160 mg/kg. HD-S10 radiotelemetric probes (Data Science International, St. Paul, Minnesota, USA) were used for direct blood pressure (BP) measurements during the 5-week post-DOXO follow up as described previously [44, 45].

### Analytical procedures and chemicals

Kidney and liver biomarkers were measured in plasma by FUJI DRI-CHEM analyser using appropriate slides (FUJIFILM Corp., Tokyo, Japan) *CRE-P III* for creatinine and *ALB-P* for albumin. Plasma levels of triacylglycerols (TAG) and total cholesterol were measured using commercially available kits (Erba Lachema, Czech Republic). Concentrations of cTnT in plasma were determined using the Elecsys Troponn T hs STAT test (Roche Diagnostics, Basel, Switzerland, detection limit of 0.003 µg/l).

Nitrate/nitrite levels were measured by a colorimetric assay (780001, Cayman Chemical, Ann Arbor, MI, USA). Commercially available ELISA kits were used to measure: urine cGMP (581021, Cayman Chemical, Ann Arbor, MI, USA); plasma and renal noradrenalin (RE59261; IBL Int., Hamburg, Germany). Sodium and potassium in plasma and urine were measured by BWB-XP flame photometer (BWB Technologies Ltd., Berkshire, UK). Detailed protocols of plasma and tissue preparation are described in our previous studies [44, 46–49].

#### Histological examination of the kidneys

The kidneys were fixed in 4% formaldehyde, dehydrated and embedded in paraffin. The sections were stained with periodic acid for Schiff reaction and evaluated in a blind-test fashion. The glomerulosclerosis index (GSI) and kidney cortical tubulointerstitial injury (TSI) were evaluated as previously described in detail in ours and other studies [50–55].

#### Histological examination of the myocardium

The transverse sections of the heart ventricles were immersed in 4% neutral formaldehyde and embedded in paraffin. Serial paraffin sections (5 μm thick) were stained with Masson’s blue trichrome. The preparations were scanned using SmartZoom® ClassRoom software (Smart In Media AG, Germany) and photomicrographs were then taken at 20x magnification of the scans.

#### RNA isolation and quantitative real-time PCR

Total RNA was isolated with TRI Reagent (Sigma–Aldrich) or RNAzol® RT (#RN190; Molecular Research Centre, Inc., Cincinnati, USA) and qRT-PCR analysis was performed as described elsewhere [36, 40, 43]. Briefly, total RNA was reversely transcribed to cDNA with a High-Capacity cDNA Reverse Transcription Kit, and qPCR analysis was performed with a QuantStudio 7 Flex Real-Time PCR System or ViiA™ 7 Real-time PCR system (Applied Biosystems, Foster City, CA, USA) using a TaqMan Fast Universal PCR Master Mix (all from Applied Biosystems, U.S.A.). Commercially available assays for gene expression analysis of *ATP2A2 (SERCA2), BAX, Bcl2, Cdkn1a (p21), Col1a1, Col3a1, Fn1, GUCY1β1* (sGC receptor beta), *Il1b, Il6, Lgals3, Nppa* (ANP), *Ryr2, SOD2* were purchased either from Applied Biosystems or from Generi Biotech, Czech Republic. Detailed description of the assays can be found in Supplementary Table S1. The relative gene expression was calculated using the Pfaffl method with TBP (TATA box binding protein) or GAPDH used as a reference genes.

#### Western blot analysis

The protein expression of sGC receptor (GUCY1β1) was performed as described in our previous studies [43, 49]. Briefly, tissue samples (kidney cortex and left ventricle) were homogenized in RIPA lysis buffer containing 50 mM Tris–HCl pH 7.4, 150 mM NaCl, 1% NP-40, 0.25% deoxycholic acid, 1 mM EDTA; supplemented with protease inhibitor cocktail (Sigma-Aldrich, St. Louis, MO, USA). Protein concentration in the supernatant was measured using Pierce BCA protein assay (Thermo Scientific, Waltham, MA, USA). In total, 50 μg (kidney cortex) or 80 μg (left ventricle) of protein was separated by sodium dodecyl sulphate polyacrylamide gel electrophoresis (SDS-PAGE) and transferred onto the polyvinyl difluoride (PVDF) membrane. Membranes were blocked with 5% non-fat dry milk in TRIS buffered saline with Tween20 (TBS-T) + 2.5% BSA for 1 h at room temperature. After washing with TBS-T, the membranes were incubated with primary antibodies overnight at 4 °C. After O/N incubation and washing, the membranes were incubated with horseradish peroxidase-conjugated secondary antibody for 1 h at room temperature. After last washing, the immunoblots were exposed to SuperSignal West Dura Substrate (Thermo Scientific, Rockford, IL, USA) for chemiluminiscent detection. Relative densitometry was determined using ImageJ software (NIH, Bethesda, MD, USA). All protein data was normalized to the housekeeping protein β-actin (kidney) and GAPDH (left ventricle). The antibodies and dilutions used were as follows: GUCY1β1 (1:1000; NBP3-16248, Novus Biologicals, LLC, CO, USA); anti-β-actin (1:7500; A5441, Sigma Aldrich, MO, USA); GAPDH-HRP (1:500; sc-25778, Santa Cruz Biotechnology, Inc., Texas, USA).

#### Statistical analysis

All values are expressed as means ± SEM. Graph-Pad Prism software (Graph Pad Software, San Diego, California, USA) was used for statistical analysis of the data. Comparison of survival curves was performed by log-rank (Mantel-Cox) test. Multiple-group comparisons were performed by multiple *t* test, Wilcoxon´s signed-rank test, one-way or two-way analysis of variance followed by the recommended post hoc test as appropriate. Values exceeding the 95% probability limits (*P* < 0.05) were considered statistically significant. The significance levels are indicated on figures with asterisks: *P* > 0.05 (NS); *P ≤ 0.05; **P ≤ 0.01; ***P ≤ 0.001; ****P ≤ 0.0001. The data and statistical analysis comply with the recommendations on experimental design and analysis in physiology and pharmacology (Curtis et al. 2018).

## Results

### Series 1—Analysis of survival and echocardiographic assessment of cardiac function in DOXO-induced model of HFrEF with 10-week post-DOXO follow up

#### Survival and body weight (Fig. 2)

**Fig. 2:**
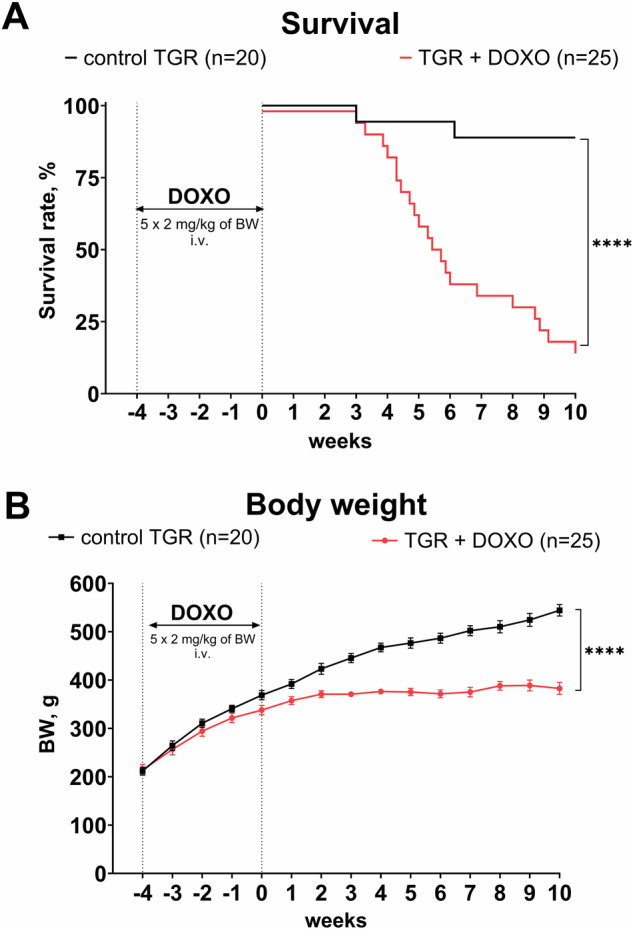
**A** Survival and (**B**) body weight (BW) changes in Ren-2 transgenic hypertensive rats (TGR) treated weekly with doxorubicin (DOXO) for 5 weeks in comparison to the control TGR (placebo-treated) group; ****P ≤ 0.0001 by log-rank Mantel-Cox (survival) and by 2-way ANOVA with repeated measures with Bonferroni’s multiple comparisons test (BW changes)

As shown in Fig. 2A, DOXO-treated TGR started to die four weeks after the last DOXO administration with a major decline in survival between the 4th and 6th week. At the end of the study (week +10) only 16% rats were still alive. Two vehicle-treated TGR died on week +3 and +6, respectively, whereas all the others survived until the end of experiment (final survival rate 89%). As shown in Fig. 2B, there was no significant difference in the initial BW between vehicle-treated and DOXO-treated TGR. In the control TGR, BW gradually increased throughout the study, however, in DOXO-treated TGR reduction of BW gain was already seen during DOXO administration (week -1) and remained so until the end of the study when BW was remarkably lower than in the control TGR (383 ± 13 vs. 544 ± 12 g, *P* < 0.0001).

#### Echocardiographic evaluation (Figs. 3–4 and Supplementary Fig. S1)

**Fig. 3:**
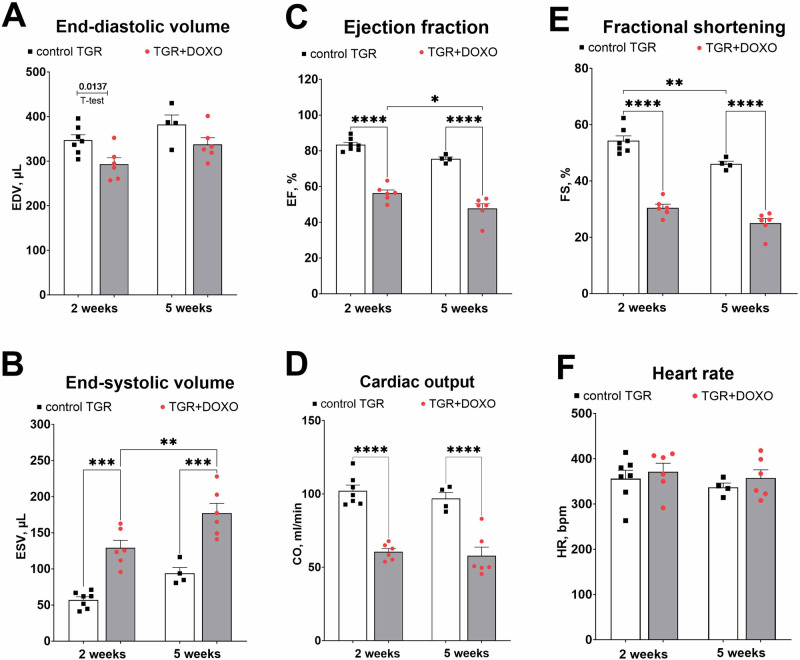
Echocardiographic evaluation (part 1) of the left ventricular parameters in Ren-2 transgenic hypertensive rats (TGR) in comparison to control TGR (placebo-treated) group, performed two and five weeks after termination of doxorubicin (DOXO) treatment (cumulative dose 10 mg/kg of BW; one injection per week for five consecutive weeks); (**A**) End-diastolic volume (EDV); (**B**) End-systolic volume (ESV); (**C**) Ejection fraction (EF); (**D**) Cardiac output (CO); (**E**) Fractional shortening (FS); (**F**) Heart rate (HR); *P ≤ 0.05; **P ≤ 0.01; ***P ≤ 0.001; ****P ≤ 0.0001 by 2-way ANOVA with repeated measures with Tukey’s multiple comparisons test

Figure 3 summarizes the evaluation of LV function by echocardiography in the very early phase (week +2) and early phase (+5) of the HFrEF development. DOXO treatment in TGR resulted in a significant decrease in LV end-diastolic volume two weeks after termination of DOXO administration, however, a significant and progressive increase in LV end-systolic volume on weeks +2 and +5 was observed in comparison with the control TGR (Fig. 3A, B). Already two weeks after the last injection of DOXO the ejection fraction (EF) of the left ventricle (LV), fractional shortening of LV (FS) and cardiac output (CO) were significantly lower than in the control TGR (Fig. 3C–F). Moreover, the decrease in EF progressed in the TGR + DOXO group and the values recorded five weeks after the last DOXO administration (48 ± 3%) were significantly lower (p < 0.05, Fig. 3F) in comparison with the values from week +2 (56 ± 2%).

**Fig. 4:**
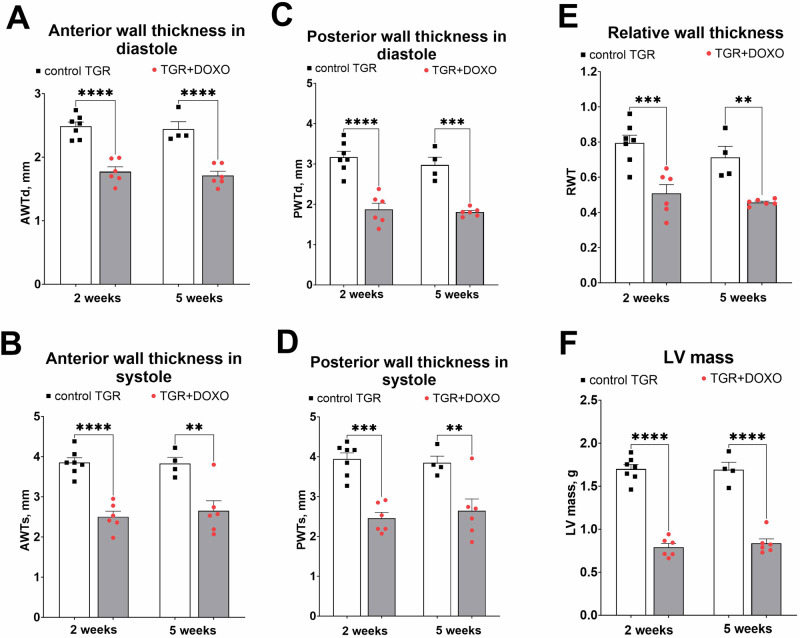
Echocardiographic evaluation (part 2) of the left ventricle parameters in Ren-2 transgenic hypertensive rats (TGR) in comparison to control TGR (placebo-treated) group, performed two and five weeks after termination of doxorubicin (DOXO) treatment (cumulative dose 10 mg/kg of BW; one injection per week for five consecutive weeks); (**A**): anterior wall thickness in diastole (AWTd). **B** Anterior wall thickness in systole (AWTs); (**C**) posterior wall thickness in diastole (PWTd); (**D**) posterior wall thickness in systole (PWTs); (**E**) relative wall thickness (RWT); (**F**) left ventricular mass (LV mass); **P ≤ 0.01; ***P ≤ 0.001; ****P ≤ 0.0001 by 2-way ANOVA with repeated measures with Tukey’s multiple comparisons test

**Fig. 5:**
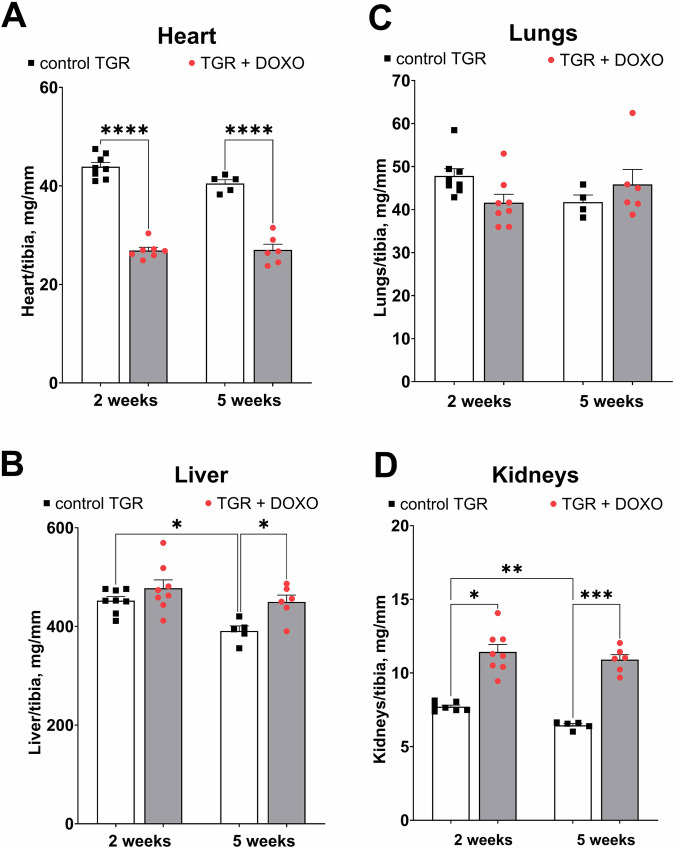
Organ weights (normalized by tibia length) after doxorubicin (DOXO) treatment (cumulative dose 10 mg/kg of BW; one injection per week for five consecutive weeks) collected two and five weeks from the last DOXO injection in ren-2 transgenic hypertensive rats (TGR) in comparison to control TGR (placebo-treated) group; (**A**) whole heart weight; (**B**) liver weight; (**C**) lungs weight; (**D**) kidney weight; * *P* ≤ 0.05; ** *P* ≤ 0.01; *** *P* ≤ 0.001; **** *P* ≤ 0.0001 by one-way ANOVA with Tukey’s multiple comparisons test

As shown in Fig. 4, DOXO administration markedly decreased LV anterior as well as posterior wall thickness, both in diastole and in systole, as compared with control TGR (Fig. 5A–D). Both the LV posterior wall thickness and LV relative wall thickness (RWT; Fig. 4E) decreased distinctly. The LV mass calculated from echocardiography parameters decreased significantly in DOXO-treated group, as seen already in the very early phase of HF development (Fig. 4F).

Echocardiographic evaluation of the right ventricle (RV) is shown in Supplementary Fig. S1. The impact of DOXO on RV was less pronounced than on LV. The fractional area change (FAC, Supplementary Fig. S1B) of the RV was significantly lower only 2 weeks after the DOXO administration in comparison with the control TGR. There was no difference in basal diameter measured in diastole, but mid diameter (after 2 weeks only) and longitudinal diameter (after 2 and 5 weeks) were significantly shorter than in the control rats (Supplementary Fig. S1A–C).

### Series 2 and 3—Evaluation of the DOXO-induced cardiac damage, liver and renal function

#### Organ weights (Fig. 5)

Figure 5A–D summarize organ weights (normalized to tibia length) two and five weeks after termination of DOXO administration. Already in the very early stage of HF (+2 week) a significant decrease in the heart weight was observed as compared with the placebo-treated counterparts. This difference was also recorded five weeks after the last DOXO injection (Fig. 5A). DOXO treatment did not have any significant impact on lung weight, only a moderate impact on liver, but increased kidney weight (Fig. 5B–D).

#### Evaluation of cardiac, liver and renal biomarkers. Fluid and electrolyte balance and activation of SNS (Figs. 6, 7; Supplementary Figs. S2, S3)

To evaluate the cardiac, liver and renal function, blood and urine samples were collected from rats in selected time points, i.e. at the weeks +2 and +5 after termination of DOXO treatment.

**Fig. 6:**
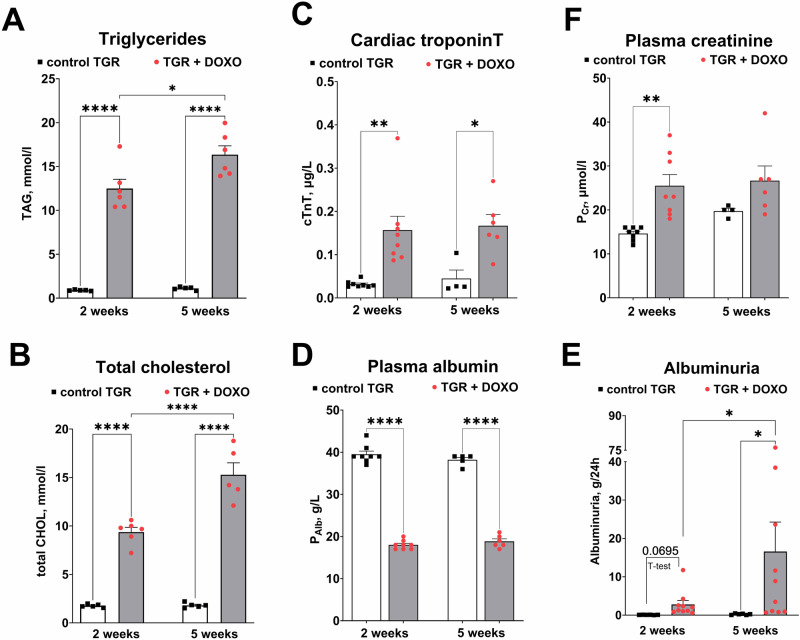
Plasma and urine parameters after doxorubicin (DOXO) treatment (cumulative dose 10 mg/kg BW; one injection per week for five consecutive weeks) measured two and five weeks after the last DOXO injection in Ren-2 transgenic hypertensive rats (TGR) in comparison to control TGR (placebo-treated) group; (**A**) Triglycerides (TAG); (**B**) total cholesterol (total CHOL); (**C**) plasma cardiac troponin T (cTnT); (**D**) plasma albumin (P_Alb_); (**F**) plasma creatinine (P_Cr_); (**E**) albuminuria; *P ≤ 0.05; **P ≤ 0.01; ****P ≤ 0.0001 by 2-way ANOVA with repeated measures with Tukey’s multiple comparisons test

The blood samples collected already at the very early phase (week +2) were showing the signs of hypertriglyceridemia: after centrifugation the remaining plasma was “milky” and opalescent, suggesting increased triglyceride content. Plasma levels of triglycerides (TAG) and total cholesterol after DOXO administration were in fact more than 10-fold higher than in the control TGR (Fig. 6A, B).

DOXO treatment in TGR markedly increased plasma cTnT levels as compared with vehicle-treated TGR, as seen already two weeks after DOXO treatment, and the increase was sustained until week +5, suggesting significant and ongoing damage to cardiomyocytes even in the post-DOXO period (Fig. 6C).

**Fig. 7:**
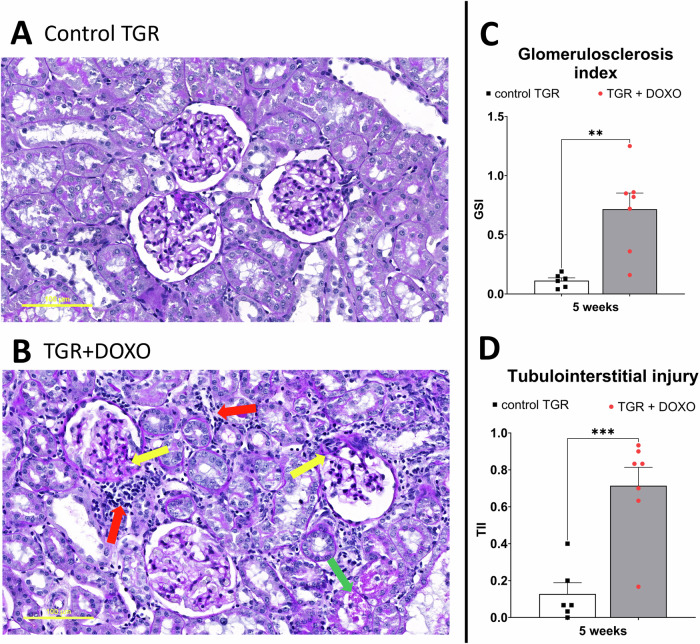
Histological examination of kidney slices stained with periodic acid Schiff (PAS) in (**A**) control TGR (placebo-treated) group and (**B**) DOXO-treated TGR animals five weeks after doxorubicin (DOXO) treatment (cumulative dose 10 mg/kg BW; one injection per week for five consecutive weeks). **C** Glomerulosclerosis index (GSI) and (**D**) tubulointerstitial injury index (TII). DOXO treated rats exhibited signs of nephrotic syndrome: focal segmental glomerulosclerosis (yellow arrows), interstitial fibrosis and inflammatory leucocyte infiltrates (red arrows) and tubular atrophy (green arrows). **P ≤ 0.01; ***P ≤ 0.001 by one-way ANOVA with Tukey’s multiple comparisons test. The scale bar in the figure is 100 µm

DOXO treatment significantly decreased plasma albumin (significant after two and five weeks, Fig. 6C). DOXO treatment increased plasma creatinine as observed two weeks after the last injection, but the difference was not statistically significant after 5 weeks (Fig. 6F), which might suggest alleviation of kidney injury. To further evaluate potential kidney damage, we measured daily albumin excretion. DOXO treatment progressively increased the excretion of albumin in urine causing severe albuminuria (Fig. 6E). Kidney histopathology revealed severe kidney damage manifested by focal segmental glomerulosclerosis (FSGS), interstitial fibrosis, inflammatory leucocyte infiltrates and tubular atrophy (Fig. 7) – all usual signs of the nephrotic syndrome. Moreover, glomerulosclerosis index (GSI) and tubulointerstitial injury index (TII) calculated five weeks after the last DOXO injection were significantly higher than in control rats treated with placebo.

As a marker of the sympathetic nervous system (SNS) activation we measured plasma levels of noradrenaline (Ne). Elevated plasma Ne levels were observed in DOXO-treated rats two weeks after DOXO treatment termination (week +2 control TGR: 0.36 ± 0.02 versus 0.76 ± 0.07 ng/ml in TGR + DOXO, *p* = 0.0002 t test, Supplementary Fig. S2A). After five weeks, a significant increase in Ne levels was observed in both groups (*i.e*. values from week +5 were significantly higher than at week +2 for each experimental group), but the difference between the values in control and TGR + DOXO group disappeared (week +5 control TGR: 1.14 ± 0.26 versus 1.33 ± 0.2 ng/ml in TGR + DOXO, NS, Supplementary Fig. S2A). Haematocrit (Ht) slightly increased between +2 and +5 weeks after DOXO treatment, but there were no differences between DOXO-treated and control rats (Supplementary Fig. S2B).

DOXO administration decreased water intake, which was reflected by diminished diuresis (Supplementary Fig. S3A, B). A substantial decrease in sodium excretion was visible already two weeks after DOXO termination, and it was progressive (the values after five weeks were even lower), suggesting a shift towards sodium retention (Supplementary Fig. S3D). Potassium excretion was decreased (significant only after five weeks from the last DOXO injection). DOXO treatment decreased plasma sodium whereas plasma potassium was higher than in untreated rats (Supplementary Fig. S3C, E).

#### Histological examination of the myocardium (Fig. 8)

**Fig. 8:**
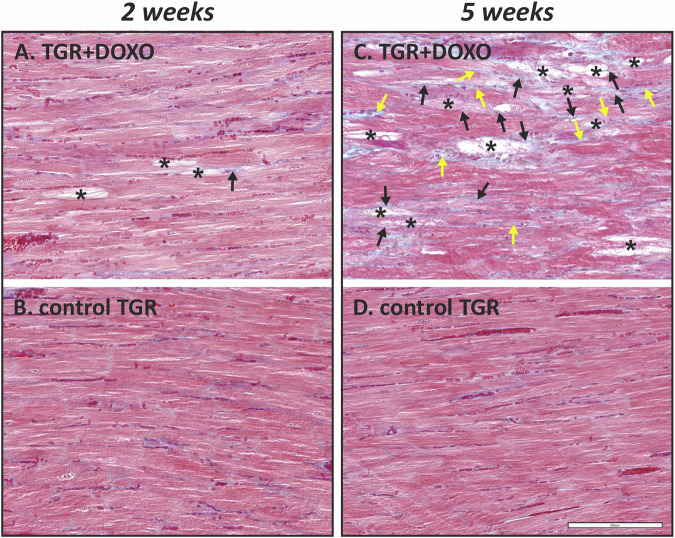
Histological examination of the myocardium in (**A**) DOXO-treated TGR animals two weeks and (**C**) five weeks after doxorubicin (DOXO) treatment (cumulative dose 10 mg/kg BW; one injection per week for five consecutive weeks) with corresponding control TGR (placebo-treated) group; – (**B**, **D**), respectively. Focal degenerative changes manifested mainly by loss of myofibrils and vacuolization of the cytoplasm of cardiomyocytes (*) were observed in both DOXO-treated groups (**A**, **C**). These changes were apparently more frequent and severe in the latter group (**C**) with longer post-treatment follow up. In this group (**C**), collagen fibre staining (blue colour) also revealed distinct DOXO-induced fibrosis—both replacement (black arrow) and perivascular/interstitial (yellow arrow), while these changes were typically rare in the DOXO group with the shorter follow up (**A**). TGR – Ren2 transgenic rats, DOXO doxorubicin. Masson’s blue trichrome staining. Bar 200 µm

In DOXO-treated TGR, focal degenerative changes in the myocardium affecting mainly the LV (including the interventricular septum) were observed (Fig. 8A, C). Loss of myofibrils and vacuolization of cytoplasm of cardiomyocytes were recognizable as typical hallmarks of DOXO toxic damage. No such changes were observed in the LV myocardium of the control TGR (Fig. 8B, D). Two weeks after the chemotherapy (Fig. 8A), the DOXO-induced changes in the myocardium were less severe, typically with few degenerating cardiomyocytes in the affected foci. Little to no fibrosis was observed in the LV myocardium as compared to the control TGR (Fig. 8B). In contrast, five weeks after the treatment, the degenerative changes in cardiomyocytes developed further to become more evident (Fig. 8C). They were also accompanied by proliferation of collagen connective tissue resulting in both perivascular/interstitial and replacement and fibrosis. These changes were absent in the myocardium of the corresponding control rats (Fig. 8D). The results of the histological examination of the LV myocardium in higher magnification are shown in Supplementary Fig. S4.

#### Evaluation of cardiac remodelling, fibrosis, apoptosis, oxidative stress and inflammation (Figs. 9, 10; Supplementary Fig. S5)

**Fig. 9:**
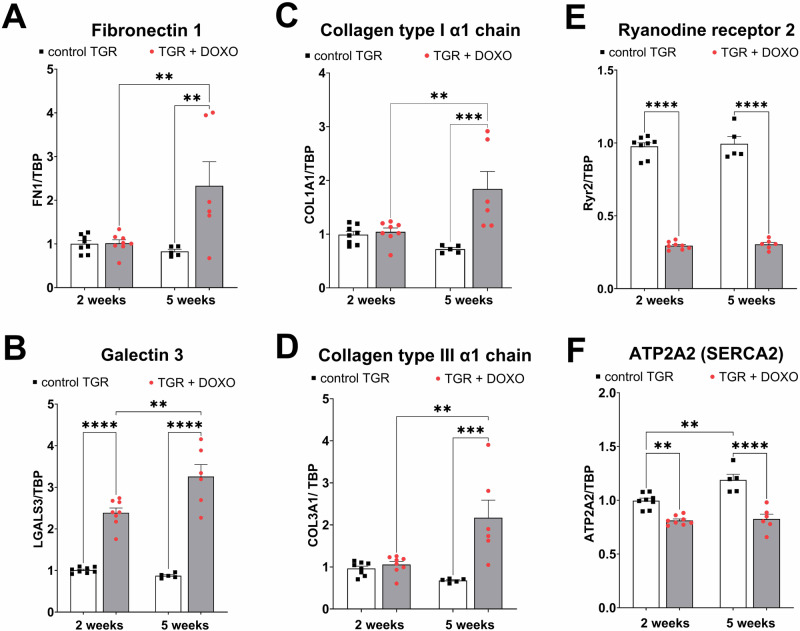
Molecular markers of myocardial remodelling, fibrosis and Ca^2+^ handling in the left ventricle after doxorubicin (DOXO) treatment (cumulative dose 10 mg/kg BW; one injection per week for five consecutive weeks) measured two and five weeks after the last DOXO injection in Ren-2 transgenic hypertensive rats (TGR) in comparison to the control TGR (placebo-treated) groups. The gene expression was determined by qRT-PCR, the data were normalized on TBP (TATA-binding protein) as a reference gene and shown relative to the control group (control TGR 2 weeks). **A** Fibronectin 1(FN1); (**B**) Galectin 3 (LGALS3); (**C**): Collagen type I α1 chain (COL1A1); (**D**) Collagen type III α1 chain (COL3A1); (**E**): Ryanodine receptor 2 (Ryr2); (**F**) ATPase sarcoplasmic/endoplasmic reticulum Ca^2+^ transporting 2 (ATP2A2/SERCA2); *P* > 0.05 (NS); *P ≤ 0.05; **P ≤ 0.01; ***P ≤ 0.001; ****P ≤ 0.0001 by one-way ANOVA with Tukey’s multiple comparisons test

To further characterise the development of HF and cardiac injury we measured relative gene expression of a spectrum of relevant proteins. Regarding the processes of cardiac remodelling and the state of tissue fibrosis, we found that gene expression of fibronectin 1 and collagen (type I and III) were elevated five weeks after the last DOXO injection (not significant after two weeks), but galectin 3 was elevated already at the very early phase of HF development (week +2; Fig. 9A–D).

The expression of the ryanodine receptor (Ryr2) through which Ca^2+^ is released from the sarcoplasmic reticulum, was markedly decreased already two weeks after the last DOXO injection and a similar change was found five weeks post-DOXO (Fig. 9E). Also, ATPase sarcoplasmic/endoplasmic reticulum Ca^2+^ transporting 2 (ATP2A2), which encodes SERCA2a—the major isoform of SERCA expressed in cardiomyocytes, was significantly decreased in expression already two weeks after last DOXO administration and this decrease was maintained at week +5 (Fig. 9F). The latter change could significantly contribute to abnormal Ca^2+^ handling and it ranks among typical findings observed in failing hearts [56].

**Fig. 10:**
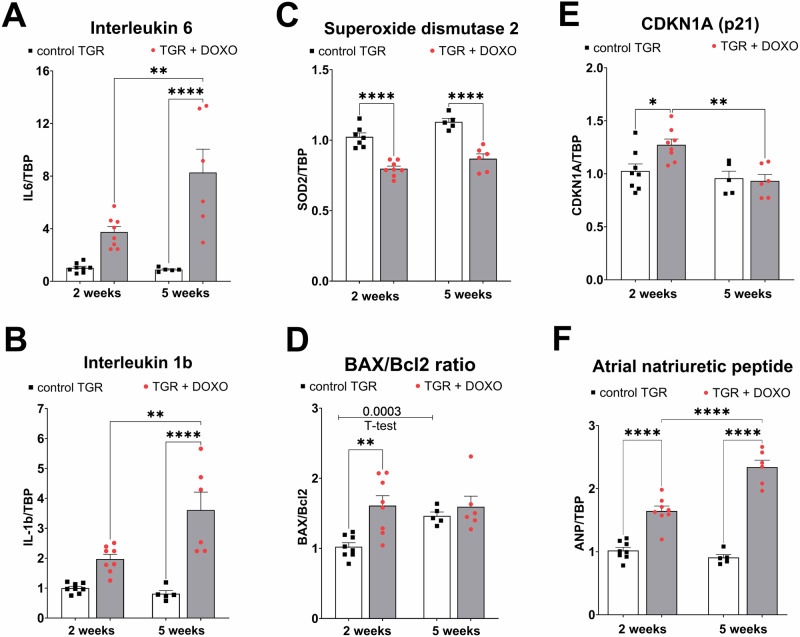
Molecular markers of myocardial inflammation, oxidative stress, apoptosis and dysfunction in the left ventricle after doxorubicin (DOXO) treatment (cumulative dose 10 mg/kg BW; one injection per week for five consecutive weeks) measured two and five weeks after the last DOXO injection in Ren-2 transgenic hypertensive rats (TGR) in comparison to the control TGR (placebo-treated) groups. The gene expression was determined by qRT-PCR, the data were normalized on TBP (TATA-binding protein) as a reference gene and shown relative to the control group (control TGR 2 weeks). **A** Interleukin 6 (IL6); (**B**) interleukin 1b (IL-1b); (**C**) superoxide dismutase 2 (SOD2); (**D**) the ratio of pro-apoptotic Bcl2 associated X (BAX) and anti-apoptotic Bcl2; (**E**) cyclin dependent kinase inhibitor 1 A (CDKN1A, p21); (**F**) atrial natriuretic peptide (ANP); *P* > 0.05 (NS); *P ≤ 0.05; **P ≤ 0.01; ***P ≤ 0.001; ****P ≤ 0.0001 by one-way ANOVA with Tukey’s multiple comparisons test

Interleukin 6 (IL6) and 1b (IL-1b) showed an increasing tendency already in the very early phase of HF development (week +2) and were significantly elevated five weeks after termination of DOXO treatment compared to untreated counterparts (Fig. 10A, B). The gene expression of superoxide dismutase (SOD2), which constitutes one of the most crucial antioxidant defence systems was already decreased two weeks after the last DOXO injection (compared to placebo-treated TGR; Fig. 10C) and it remained the same also at week +5.

Gene expression of BCL2 associated X (BAX), a pro-apoptotic regulator, was elevated in the DOXO group only after five weeks in comparison with the control group (Supplementary Fig. S5A). The expression of anti-apoptotic Bcl2 declined significantly after DOXO treatment at week +2 in comparison with the control group (Supplementary Fig. S5B). Significant decrease in Bcl2 expression was also found within control group (value at week +2 versus value + 5 week). The resulting BAX/Bcl2 ratio determined in individual animals increased significantly in the DOXO group two weeks after the treatment, while at five weeks no significant change to the corresponding control group was observed (Fig. 10D).

The gene expression of cyclin dependent kinase inhibitor 1 A (CDKN1A, p21) was also significantly higher two weeks after the last DOXO injection in comparison to the control rats. But after five weeks CDKN1A expression decreased to the level observed in the control group (the values at week +5 were significantly lower than those obtained at week +2, but they were not different to the corresponding control group; Fig. 10E).

The expression of the gene encoding atrial natriuretic peptide (ANP) was elevated two weeks after the last DOXO injection and the increase was progressive: the values determined at week +5 were not only higher in comparison to age-matched control rats (1.52 ± 0.01-fold higher; *p* = 0.0006 by one way ANOVA and Tukey’s multiple comparisons test), but there was also a significant increase in comparison with DOXO treated animals at week +2 (Fig. 10D).

#### Evaluation of NO/sGC/cGMP pathway (Fig. 11)

**Fig. 11:**
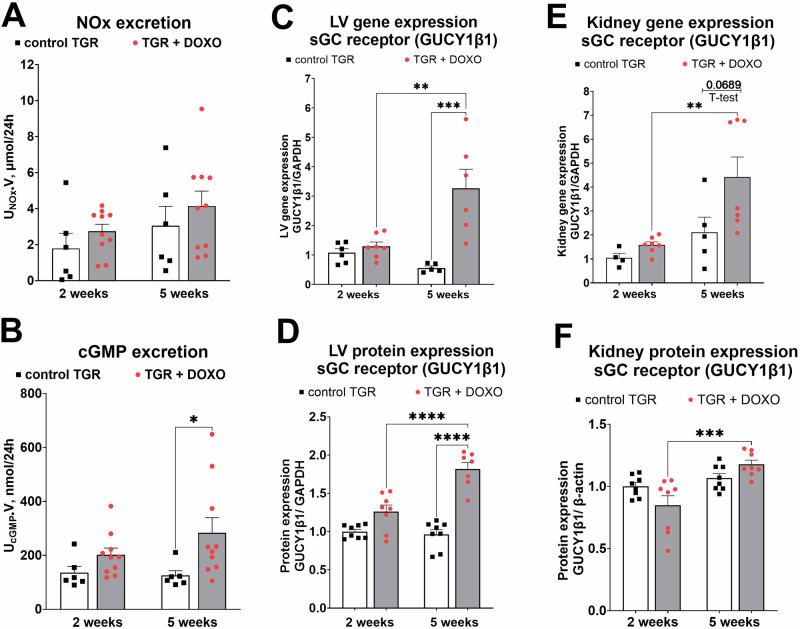
Evaluation of parameters related to NO/sGC/cGMP pathway after doxorubicin (DOXO) treatment (cumulative dose 10 mg/kg BW; one injection per week for five consecutive weeks) measured two and five weeks from the last DOXO injection in Ren-2 transgenic hypertensive rats (TGR) in comparison to control TGR (placebo-treated) groups; (**A**) excretion of nitric oxide metabolites (NOx) in urine; (**B**) excretion of cGMP in urine; (**C**) Left ventricular (LV) gene expression of sGC receptor unit β1 (GUCY1β1) determined by qRT-PCR; (**D**) LV protein expression of GUCY1β1 determined by Western Blotting; (**E**) Kidney gene expression of GUCY1β1 determined by qRT-PCR; **F** Kidney protein expression of GUCY1β1 determined by Western Blotting; *P* > 0.05 (NS); *P ≤ 0.05; **P ≤ 0.01; ***P ≤ 0.001; **** *P* ≤ 0.0001 by one-way ANOVA with Tukey’s multiple comparisons test

To evaluate the state of activation of NO/sGC/cGMP pathway, one of the most crucial system for cardiovascular and renal homoeostasis, we measured the excretion of NO metabolites (NOx: nitrate and nitrite), still considered as a good marker of NO production [57, 58]. There was only some increasing tendency (but with no significant changes) in NOx excretion (Fig. 11A). We measured urinary excretion of cGMP, which we recently proved to be a reliable marker of endogenous cGMP production, particularly in HF model [55]. DOXO treatment slightly increased cGMP excretion when measured two weeks after the last DOXO injection, and increased it significantly already after five weeks (Fig. 11B). To further characterise the NO/sGC/cGMP pathway we measured gene and protein expression of GUCY1β1. This gene encodes the beta subunit of the soluble guanylate cyclase (sGC) which catalyses the conversion of GTP (guanosine triphosphate) to cGMP (cyclic guanosine monophosphate) after NO stimulation. In both heart and kidney tissues there was a progressive increase in the expression of GUCY1β1 (on the gene and protein level), especially pronounced five weeks after termination of DOXO treatment (Fig. 11C–F). The WB membranes are shown in supplementary material (Figs. S6, S7).

### Series 4—Evaluation of the effects of DOXO on blood pressure (Fig. 12)

**Fig. 12:**
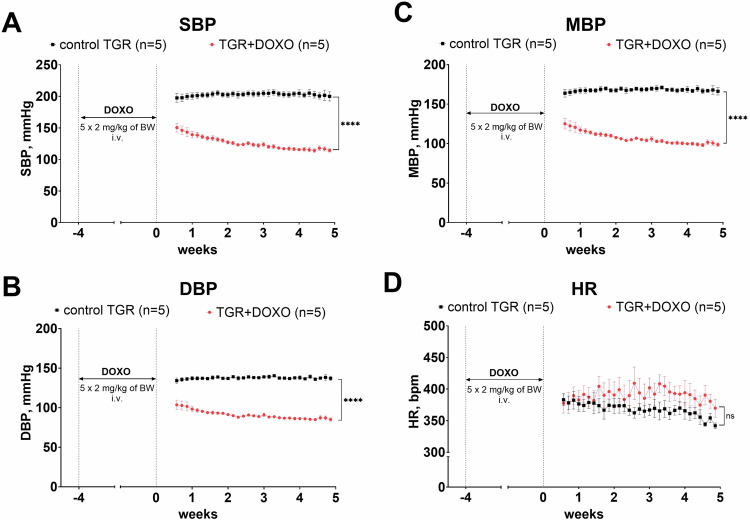
Blood pressure and heart rates after doxorubicin (DOXO) treatment (cumulative dose 10 mg/kg BW; one injection per week for five consecutive weeks) in Ren-2 transgenic hypertensive rats (TGR) in comparison to control TGR (placebo-treated) group; (**A**) systolic blood pressure (SBP); (**B**) diastolic blood pressure (DBP); (**C**) mean blood pressure (MBP); (**D**) heart rates (HR); *P* > 0.05 (NS); ****P ≤ 0.0001 by 2-way ANOVA with repeated measures with Bonferroni’s multiple comparisons test

Blood pressure was monitored by telemetry and already one week after the last DOXO injection, the BP of TGR was much lower than in the control TGR (both SBP and DBP; Fig. 12A, B). DOXO treatment tended to increase heart rate (HR), however, no statistically significant differences were observed between DOXO-treated and control TGR (Fig. 12D).

## Discussion

The main goal of the present study was to develop and characterise a small animal model of chemotherapy-induced HFrEF and cardiorenal syndrome with concomitant renin–angiotensin system (RAS) overactivation (as a contributing factor), a model which would be suitable for translational HF studies with long-term follow up. Such valuable experimental tools are currently scarce or unavailable [22–24]. To achieve this goal, a widely used chemotherapeutic drug (DOXO) was employed using the clinically relevant (intravenous) route of administration and low doses (both individual and cumulative). Our crucial finding is that already two weeks after DOXO treatment TGR showed relative bilateral cardiac atrophy and significant impairment of cardiac function with only limited impact on the function of other organs, such as the liver or lungs.

Already at the very early stage of HF development (week +2), DOXO-treated TGR showed a substantial decrease in heart weight, with marked decreases in both anterior and posterior LV wall thickness. These findings suggest that DOXO-treated TGR might be considered to display chamber remodelling described as “eccentric chamber atrophy”. DOXO also induced damage in the myocardium of the LV and interventricular septum with pattern typical for chronic DOXO cardiotoxicity (see the results of histological examination). We have also measured the concentration of cardiac troponin T in plasma (cTnT), a recognized biomarker for the degree of cardiac damage, specifically a marker of DOXO-induced cardiac injury in laboratory animals [25, 59, 60]. Already in the very early phase of HFrEF the level of cTnT was significantly elevated, suggesting damage and necrosis of cardiomyocytes, which is in accordance with the clinical data [61]. Indeed, we found impaired systolic function (reduced LVEF and LV fractional shortening) in DOXO-treated TGR by echocardiographic examination, along with reduced end-systolic LV volume, stroke volume and cardiac output. The presence of LV dysfunction at this stage was also confirmed by increased expression of ANP in the LV myocardium. Progressive decline in systolic and mean blood pressure, observed during first two weeks after DOXO-treatment, was most likely determined by decreased cardiac output.

LV dysfunction described in our model may be a result of degeneration and loss of myofibrils in cardiomyocytes and cardiomyocyte cell death, however, molecular alterations in mechanisms important for contractile function may be also involved. In particular, a disruption of intracellular calcium (Ca^2+^) homoeostasis has been described in DOXO cardiotoxicity [62]. We measured the expression of crucial genes responsible for cardiac Ca^2+^ handling in cardiomyocytes (i.e. *Ryr2* and SERCA2), and found their severe down-regulation in the myocardium of DOXO-treated TGR, which is in line with chronic DOXO effects in different animal species [25, 40, 56, 63]. This suggests impairment of both systolic and diastolic functions of the heart, which confirms previous reports on the important role of calcium in DOXO-induced cardiotoxicity [56, 63].

Regarding the other molecular events apparent at this stage of cardiotoxicity and HF development, we found DOXO-induced down-regulation of SOD2 gene expression which is essential for antioxidant defence of the mitochondria. This is another typical feature of DOXO-induced cardiac dysfunction observed in rodent and non-rodent models [25, 64] and it has been mechanistically associated with DOXO triggered and p53-mediated signalling [65].

DOXO treatment had a relatively different impact on function of the left and right ventricle. Echocardiographic examination showed that DOXO treatment caused RV atrophy in TGR, but RV fractional area change (FAC), an index of RV ejection fraction, was only slightly diminished, suggesting only slight RV impairment. This is in agreement with the histological analysis where toxic damage was found mainly in the myocardium of the LV and interventricular septum while RV myocardium was less affected (data not shown). These findings are in accordance with those reported by previous animal and human studies [66–68], although some clinical data suggest RV dysfunction as well [69].

The signs of cardiac atrophy were found to be persistent as documented by the heart weight and echocardiography data at a later time-point of the study (week +5), but there was no further progression as compared with the previous study interval (week +2). Regarding LV function change between +2 and +5 weeks as determined by echocardiography, there was a slight but significant increase in end-systolic volume and decrease in the EF after DOXO treatment, whereas other parameters showed no significant progression. Correspondingly, there was only small change in systolic and mean blood pressure at this stage of the study as determined by telemetric measurements. Gene expression of *Ryr2* and SERCA2 in the LV myocardium of DOXO-treated animals remained downregulated to the same extent in both study time-points. This all indicates that there was rather little further worsening of DOXO-induced systolic dysfunction at this stage of the study. However, gene expression of ANP in the LV myocardium suggests moderate worsening of the LV wall stress.

Differences have been found between the histological features of the LV myocardium of DOXO-treated animals at very early (week +2) and early (week +5) post-treatment follow up. The degenerative changes to cardiomyocytes were more often seen in the myocardium at the later time-point and they were usually accompanied by apparent fibrosis, which was only rarely seen in the earlier phase of the study (week +2). This is in accordance with a significant increase of the LV expression of collagen I and III, fibronectin 1 and galectin 3 observed at this stage. Furthermore, progressive increase in the LV expression of inflammatory markers (IL1B and IL6) was observed at the same time. These observations implicate progression of morphological damage to cardiomyocytes and concomitant changes in extracellular matrix and inflammatory response. Gene expression of pro-apoptotic BAX was elevated in LV myocardium, but only in the early phase, i.e. five weeks after cessation of DOXO treatment. In the very early phase (week +2) the expression of BAX was not significantly affected, but Bcl2 expression was significantly downregulated at this time point. As a result, the BAX/Bcl2 ratio, which is used as an indicator of apoptotic signalling, increased significantly in the LV after DOXO treatment at week +2. Thus, our results support previous findings on the important role of apoptosis in DOXO toxicity and development of DOXO-induced cardiomyopathy with subsequent development of chemotherapy-induced HFrEF.

Another experimental application of DOXO is to induce nephrotic syndrome (NS) in rodents [8, 70–72]. NS is a prevalent renal disorder characterized by severe proteinuria, hyperlipidaemia, hypoalbuminemia and oedema [73]. DOXO-induced NS in rats closely resembles renal injury in patients with focal segmental glomerulonephritis [8]. In our model we have observed all typical signs of NS, i.e. increased cholesterol level (hyperlipidaemia), increased albuminuria and decreased plasma albumin content (hypoalbuminemia). Moreover, renal histopathology also confirmed typical changes observed in NS.

Hypoalbuminemia in NS is usually a result of extreme proteinuria and not of impaired albumin synthesis in the liver. In our studies liver weight was only slightly increased (recorded only after five weeks). Taken together, we can suggest that the synthetic liver function was preserved. Moreover, our low dose chemotherapy-induced HFrEF model with cardiorenal syndrome shows several advantages over the common approach with DOXO administered *i.p*. in relatively high cumulative dose in rodents [37, 38]. First, by employing the *i.v*. route we eliminated the local injury of the peritoneum, one of the major drawbacks of the *i.p*. protocol [35, 36]. Second, we reduced the cumulative dose of DOXO to 10 mg/kg of BW. The cardiotoxicity of DOXO is known to be dose-dependent and ultimately leads to HF and irreversible cardiac dysfunction [74]. The dosage of 400, 500, and 550 mg/m^2^ were estimated to correspond with 5%, 16%, and 26% occurrence of heart failure in cancer patients, respectively [74].

Taken together, our modified model of HFrEF with cardiorenal syndrome exhibits myocardial damage typical for DOXO-related cardiotoxicity and nephrotic syndrome, but only a degree of initial and transient liver function impairment. There was no other critical organ injury, which corresponds well with a clinical situation of cancer patients receiving anthracycline therapy [1–3, 5, 75]. Since cancer patients with chemotherapy-induced HFrEF exhibit transient liver and kidney impairment and in long term perspective renal failure can complicate HFrEF, our current model resembles the relevant clinical condition [1–3, 5, 75]. One of the advantages of the present transgenic animal model can be the presence of RAS overactivation and distinct hypertension, both of which can markedly accelerate development of HFrEF and NS during and after DOXO treatment. Hence, it is possible to study the pathological events which occur in intact wild type animals after higher cumulative doses of DOXO, but often at the expense of general nonspecific toxicity burden, and/or after longer follow up.

Furthermore, we measured the excretion of nitric oxide metabolites (NOx) and cyclic guanosine monophosphate (cGMP) in urine, both belonging to NO/sGC/cGMP complex, which is one of the major signalling cascades for regulation of cardiovascular, cardiopulmonary and cardiorenal function [76, 77]. Apart from direct vasodilatation, the NO/sGC/cGMP signalling exerts anti-fibrotic and anti-proliferative actions and influences inflammatory processes [78, 79]. Considering this critically important role of the NO/sGC/cGMP in the cardiorenal physiology and pathophysiology, targeting this pathway is currently considered as one of the most promising approaches to HFrEF treatment [77, 80, 81] and could have a large potential also for the treatment of chemotherapy-induced HF. We found that the excretion of cGMP in urine was significantly elevated no sooner than five weeks after termination of DOXO treatment. This unexpected result inspired us to further investigate the key players in this pathway. Therefore, we measured gene and protein expression of GUCY1β1 which encodes β-subunit of sGC receptor, described as essential for sGC function [82]. Both in the kidneys and in the LV we found that the expression of β-subunit of sGC receptor was elevated, both on gene and protein level.

In our previous studies we found increased cGMP excretion after induction of HF by aorto-caval fistula (ACF) [55]. Our previous and current data suggest that activation of NO/sGC/cGMP pathway, both in ACF- and DOXO-induced HF, exemplifies activation of the endogenous, vasodilatory/natriuretic systems as a part of protective defence strategy of the body to alleviate the development of HF. Therefore, the findings further support the paradigm for development of new therapeutic strategies for HF (all phenotypes) that emerged after the success of neprilysin inhibition [16–18, 83]. Evidently, apart from blocking harmful effects of neurohormonal systems (e.g. sympathetic nervous system), augmentation of other potentially protective pathways should be considered. It was already proposed that targeting the NO/sGC/cGMP pathway can attenuate chemotherapy-induced cardiotoxicity and even potentially reduce the occurrence of secondary malignant tumours that might develop after DOXO-treatment [84]. Based on the present results, we suggest that boosting the NO/sGC/cGMP pathway could be a new therapeutic target to limit chemotherapy-induced HFrEF.

### Limitations of the study

One of the limitations of our study is that it is difficult to clearly differentiate between early and late stages of DOXO cardiotoxicity, whereas such differentiation is clearly established in human medicine. The unique transgenic background involving RAS overactivation and distinct hypertension make TGR markedly more sensitive to DOXO treatment and the development of HF is thus highly accelerated. This also explains why the survival study showed relatively high mortality after DOXO, despite the lowered cumulative dose. Moreover, the life span of rats and humans is markedly different. It was estimated that rats live around 27 times “faster” than people: one human year is an equivalent of almost 2 weeks in rats [85, 86]. However, such calculations cannot be fully relied on in interpretation, even in intact wild type animals. Another limitation of our study is that our model does not include any oncologic malignancy. It is known that the presence of tumours in the organism as well as indirect consequences of their treatment might contribute to the deterioration of cardiac function. Admittedly, technical limitations also did not allow to include some valuable analyses which might be useful for the translational perspective of this model, such as NT-proBNP/BNP determination, and load-independent examination of cardiac function, such as invasive pressure-volume analysis, and morphometric analysis of the myocardium.

## Conclusion

In summary, our model of chemotherapy-induced HFrEF with cardiorenal syndrome obtained by intravenous administration of DOXO in hypertensive rats with Ren2 overexpression may be considered to mimic cardiac chamber remodelling close in character to the “eccentric chamber atrophy”. DOXO administration elicited myocardial damage typical for DOXO-related cardiotoxicity and nephrotic syndrome, without major damage of the peritoneum, lungs and liver. The new model is optimal for studying the pathophysiology of chemotherapy-induced HFrEF and concomitant renal failure, and for evaluation of long-term effectiveness of new therapeutic strategies, which is important because the current therapies are still insufficiently effective for this form of HF.

Based on previous and current data showing DOXO-induced activation of NO/sGC/cGMP system, we propose that augmentation of potentially protective endogenous pathways, such as NO/sGC/cGMP system, should be considered as a new therapeutic target for chemotherapy-induced HFrEF.

## Supplementary information

Supplementary information
