# Supplementary material for: Characterization of a new model of chemotherapy-induced heart failure with reduced ejection fraction and nephrotic syndrome in Ren-2 transgenic rats
Source: Hypertens Res. 2024 Sep 9;47(11):3126–46. doi: 10.1038/s41440-024-01865-7 (PMC11534684; doi:10.1038/s41440-024-01865-7)
Supplement: Supplementary file 1 — Supplementary information [file 41440_2024_1865_MOESM1_ESM.docx]

**Supplementary Table S1.** **Summary of gene expression assays used for reverse transcription quantitative real-time PCR**

^#^Individual gene expression assays were obtained from Applied Biosystems (AB, USA) or Generi Biotech (GB, Czech Republic)

| Gene (protein) symbol | Gene (protein) name | # | qPCR assay | Sequence for design | Localization |
| --- | --- | --- | --- | --- | --- |
| *Atp2a2* (SERCA2) | ATPase, Ca^2+^ transporting, cardiac muscle, slow twitch 2 | AB | Rn00568762_m1 | NM_001110139.2  NM_001110823.2  NR_027839.1 | exon5 / exon6 |
| *Bax* | BCL2 associated X, apoptosis regulator | AB | Rn01480161_g1 | NM_017059.2 | exon5 / exon6 |
| *Bcl2* | BCL2 apoptosis regulator | AB | Rn99999125_m1 | NM_016993.1 | exon1 / exon2 |
| *Cdkn1a* (p21) | Cyclin dependent kinase inhibitor 1A | AB | Rn00589996_m1 | NM_080782.3  XM_006256128.2 | exon2 / exon3 |
| *ol1a1* | Collagen, type I, alpha 1 | AB | Rn01463848_m1 | NM_053304.1 | exon1 / exon2 |
| *Col3a1* | Collagen, type III, alpha 1 | GB | rCOL3A1_Q1 | NM_032085.1 | exon48 / exon49 |
| *Fn1* | Fibronectin 1 | GB | rFn1_Q1 | NM_019143.2 | exon41 / exon42 |
| *Gapdh* | Glyceraldehyde-3-phosphate dehydrogenase | AB | Rn01775763_g1 | NM_017008.4 | exon8 |
| *Gucy1b3* | Guanylate cyclase 1, soluble, beta 3 (Gucy1b1, SGC) | AB | Rn00562775_m1 | NM_012769.2 | exon3 / exon4 |
|  |  |  |  |  |  |
| *Il1b* | Interleukin 1 beta | AB | Rn00580432_m1 | NM_031512.2 | exon5 / exon6 |
| *Il6* | Interleukin 6 | AB | Rn99999011_m1 | NM_012589.2 | exon6 / exon7 |
| *Lgals3* | Lectin, galactoside-binding, soluble (Galectin 3) | AB | Rn04219572_m1 | NM_031832.1 | exon2/ exon3 |
| *Nppa* (ANP) | Natriuretic peptide A (Atrial natriuretic peptide) | AB | Rn00664637_g1 | NM_012612.2 | exon1 / exon2 |
| *Ryr2* | Ryanodine receptor 2, cardiac | AB | Rn01470303_m1 | NM_001191043.1, NM_032078.2 | exon86 / exon87  exon87/ exon 88 |
| *Sod2* | Superoxide dismutase 2, mitochondrial | AB | Rn00690588_g1 | NM_017051.2 | exon3 / exon4 |
| *Tbp* | TATA box binding protein | AB | Rn01455646_m1 | NM_001004198.1 | exon4 / exon5 |

In genes, in which the protein symbol/name is not the same as the gene's, the protein symbol/name is mentioned in brackets.

# SUPPLEMENTARY FIGURES


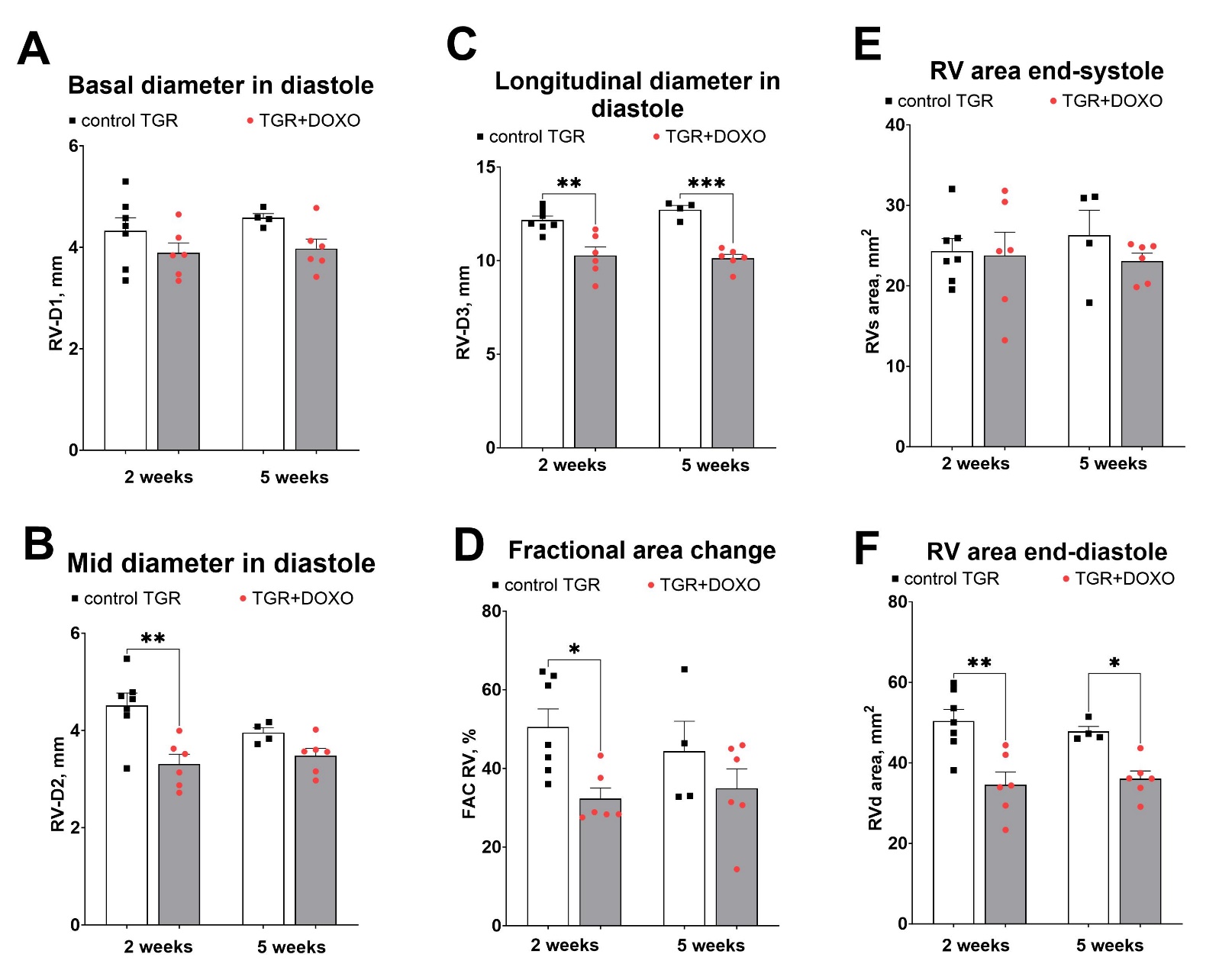


**Figure S1. Echocardiographic evaluation of the right ventricle (RV) parameters** in Ren-2 transgenic hypertensive rats (TGR) in comparison to control TGR (placebo-treated) group performed two and five weeks after termination of doxorubicin (DOXO) treatment (cumulative dose 10 mg/kg of BW; one injection per week for five consecutive weeks); **A**: Basal diameter in diastole (RV-D1); **B:** Mid diameter in diastole (RV-D2); **C:** Longitudinal diameter in diastole (RV-D3); **D:** Fractional area change (FAC RV); **E:** RV area in end-systole (RVs area) **F:** RV area in end-diastole (RVd area); *P≤0.05; ** P≤0.01; *** P≤0.001; **** P≤0.0001 by 2-way ANOVA with repeated measures with Tukey's multiple comparisons test.


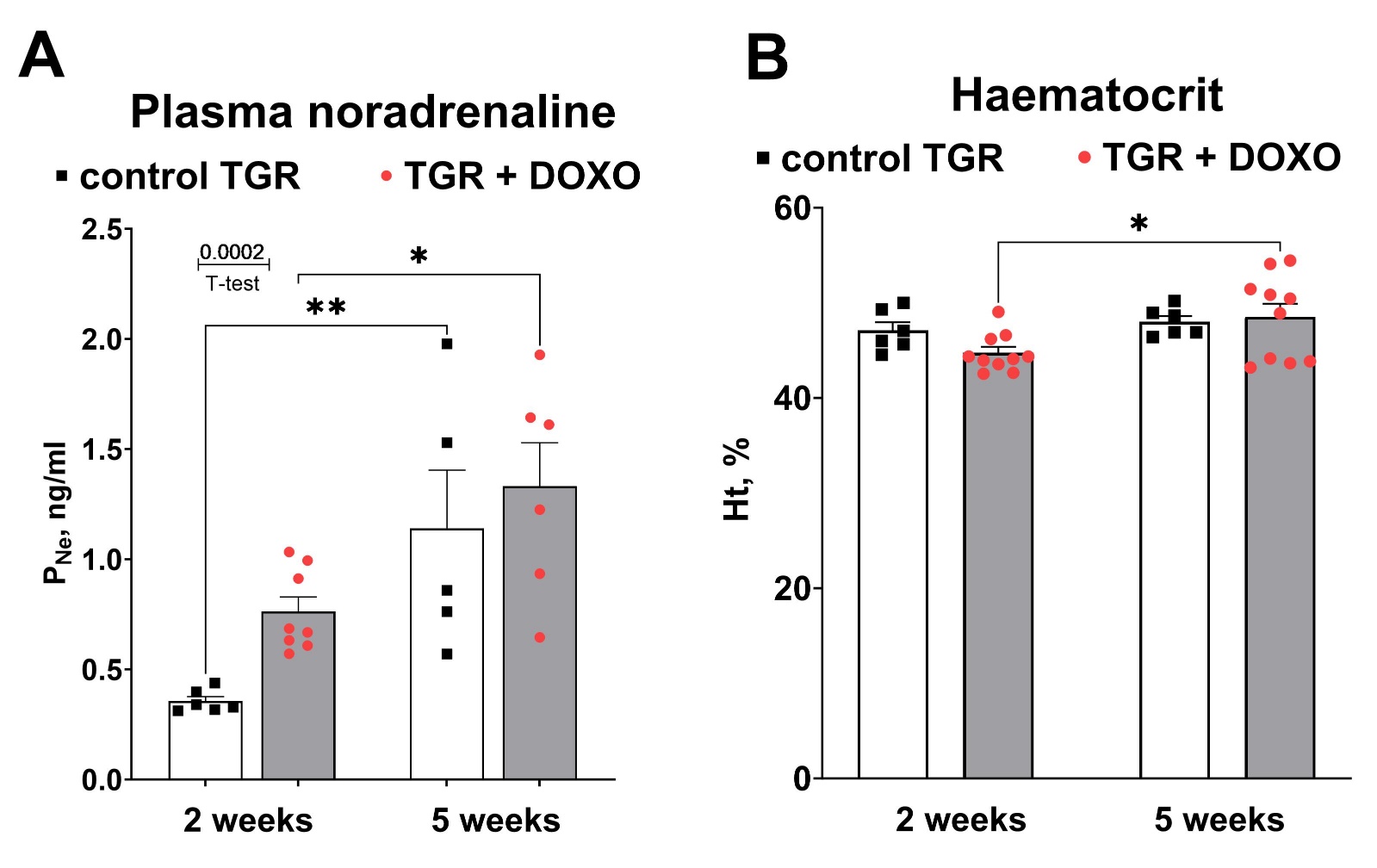


**FIGURE S2. A:** Plasma noradrenaline (P_Ne_) and **B:** haematocrit (Ht) after doxorubicin (DOXO) treatment (cumulative dose 10 mg/kg BW; one injection per week for five consecutive weeks) measured two and five weeks after the last DOXO injection in Ren-2 transgenic hypertensive rats (TGR) in comparison to control TGR (placebo-treated) group; *P≤0.05; ** P≤0.01; by 2-way ANOVA with repeated measures with Tukey's multiple comparisons test.


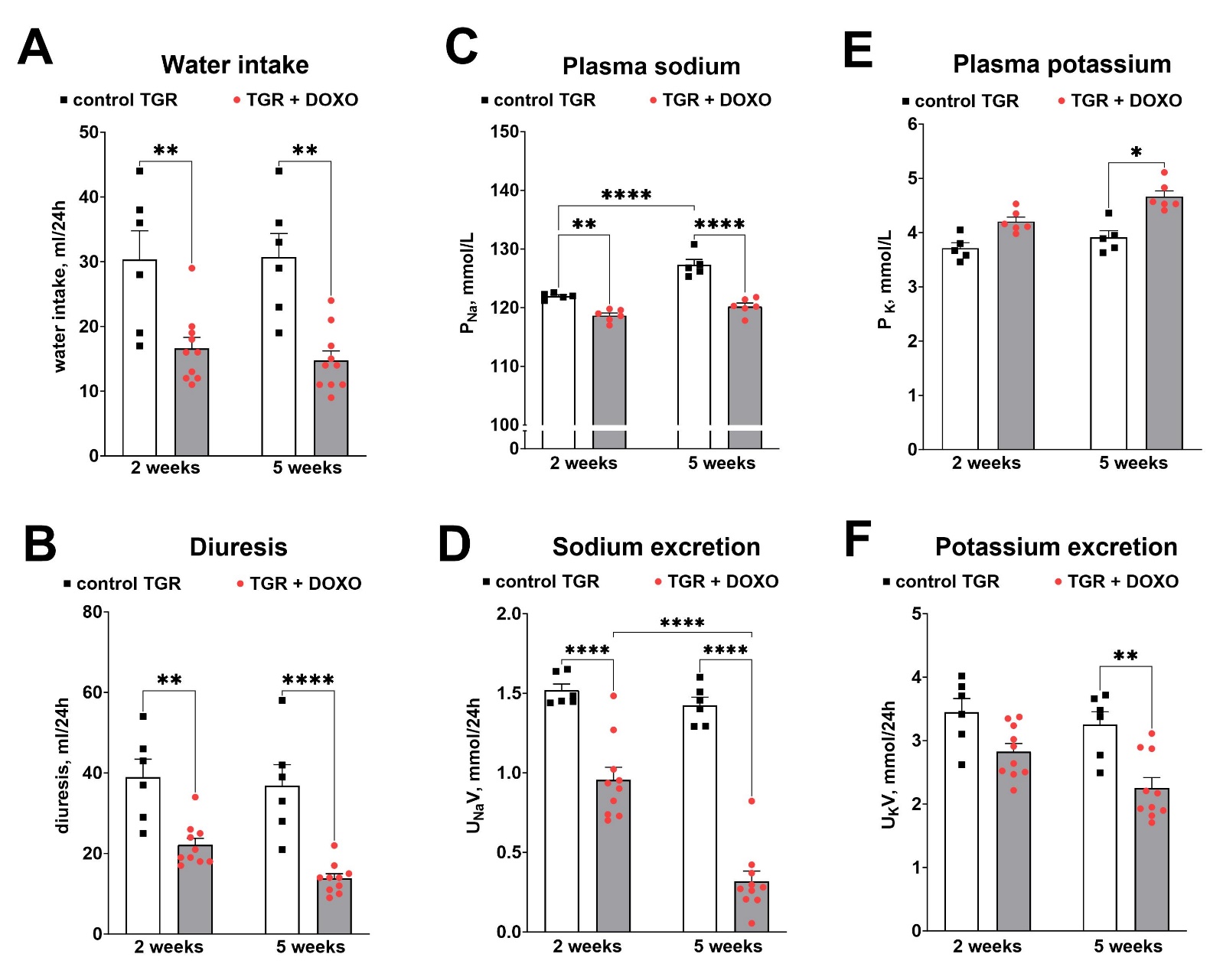


**FIGURE S3.** **Fluid and electrolyte balance** in Ren-2 transgenic hypertensive rats (TGR) after doxorubicin (DOXO) treatment (total dose 10mg/kg of BW; one injection per week for five consecutive weeks) after 2 and 5 weeks from the last DOXO injection in comparison to control TGR (placebo treated) group; **A:** water intake; **B:** diuresis; **C:** plasma sodium concentration (P_Na_); **D:** sodium excretion (U_Na_V); **E:** plasma potassium concentration (P_K_); **F:** potassium excretion (U_K_V); P>0.05 (NS); * P≤0.05; ** P≤0.01; *** P≤0.001; **** P≤0.0001 by 2way ANOVA with repeated measures with Tukey's multiple comparisons test.


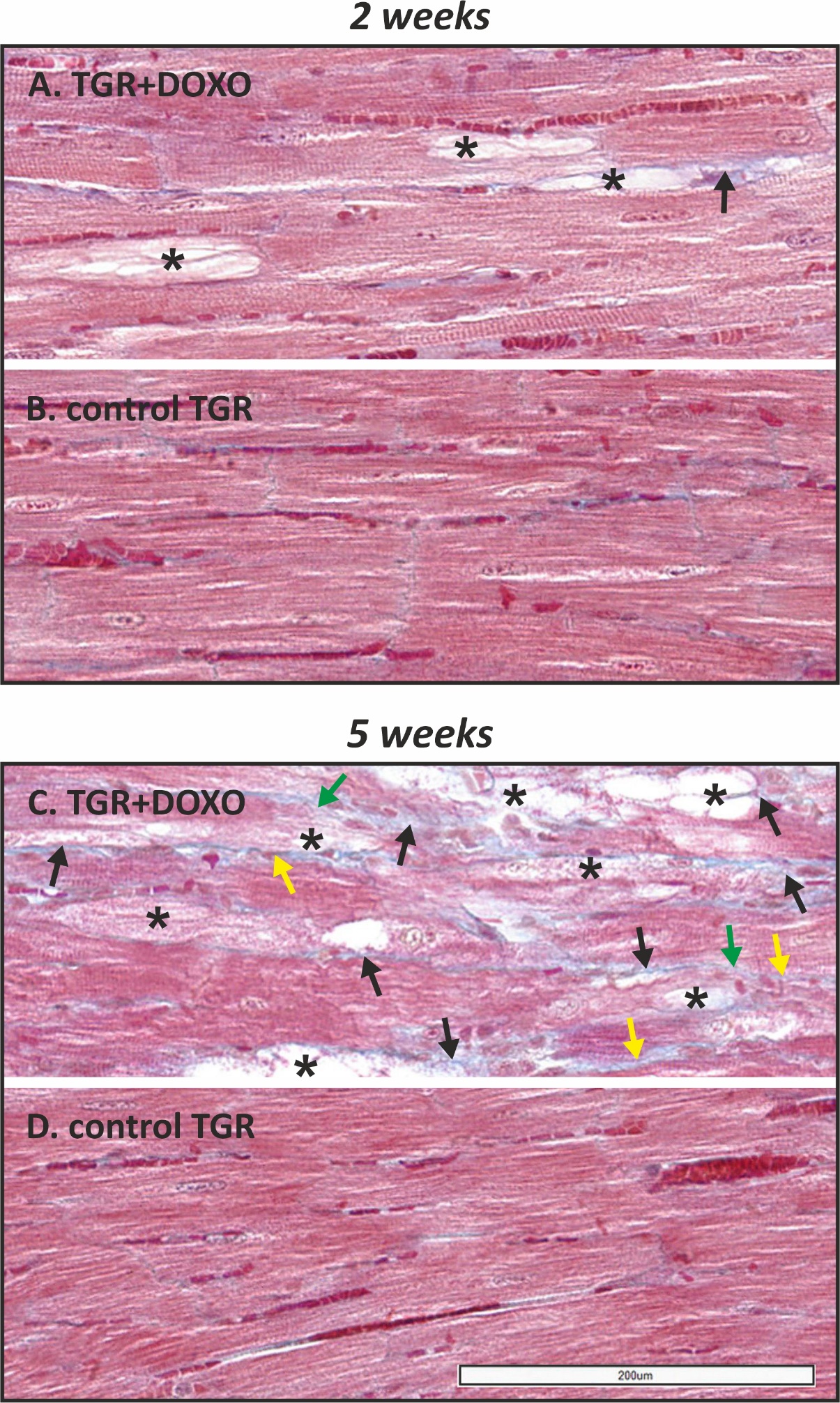


**Figure S4. The light microscopy examination of the left ventricular myocardium in higher magnification.** The focal degenerative changes manifested mainly by loss of myofibrils and vacuolization of the cytoplasm of cardiomyocytes (*). The interstitial fibrosis (yellow arrow) represented the source of connective tissue for the replacement fibrosis (black arrow) with a gradual transition between the two forms (green arrow). TGR – Ren2 transgenic rats, DOXO – doxorubicin.  Masson's blue trichrome staining. Bar 200 µm.


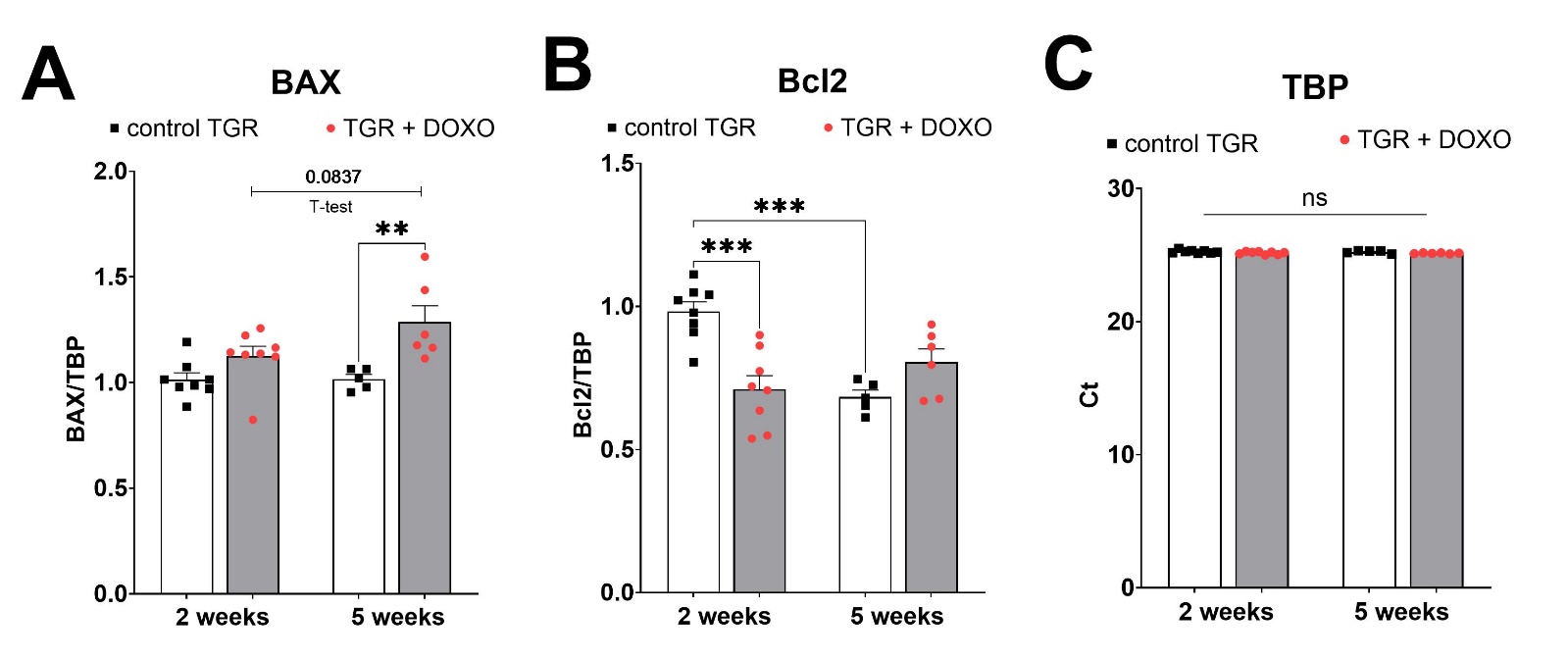


**FIGURE S5. Gene expression of A:** Bcl2 associated X (BAX) and **B:** Bcl2 in LV collected from Ren-2 transgenic hypertensive rats (TGR) after doxorubicin (DOXO) treatment (cumulative dose 10 mg/kg BW; one injection per week for five consecutive weeks) two and five weeks after the last DOXO injection in comparison to control TGR (placebo-treated). Data were normalized on TBP (TATA box binding protein) and expressed relative to the control group (control TGR 2 weeks). **C:** The stability of the housekeeping gene TBP (TATA box binding protein) in the study arms is shown as Ct values in individual animals; ** P≤0.01; *** P≤0.001; by one-way ANOVA with Tukey's multiple comparisons test.


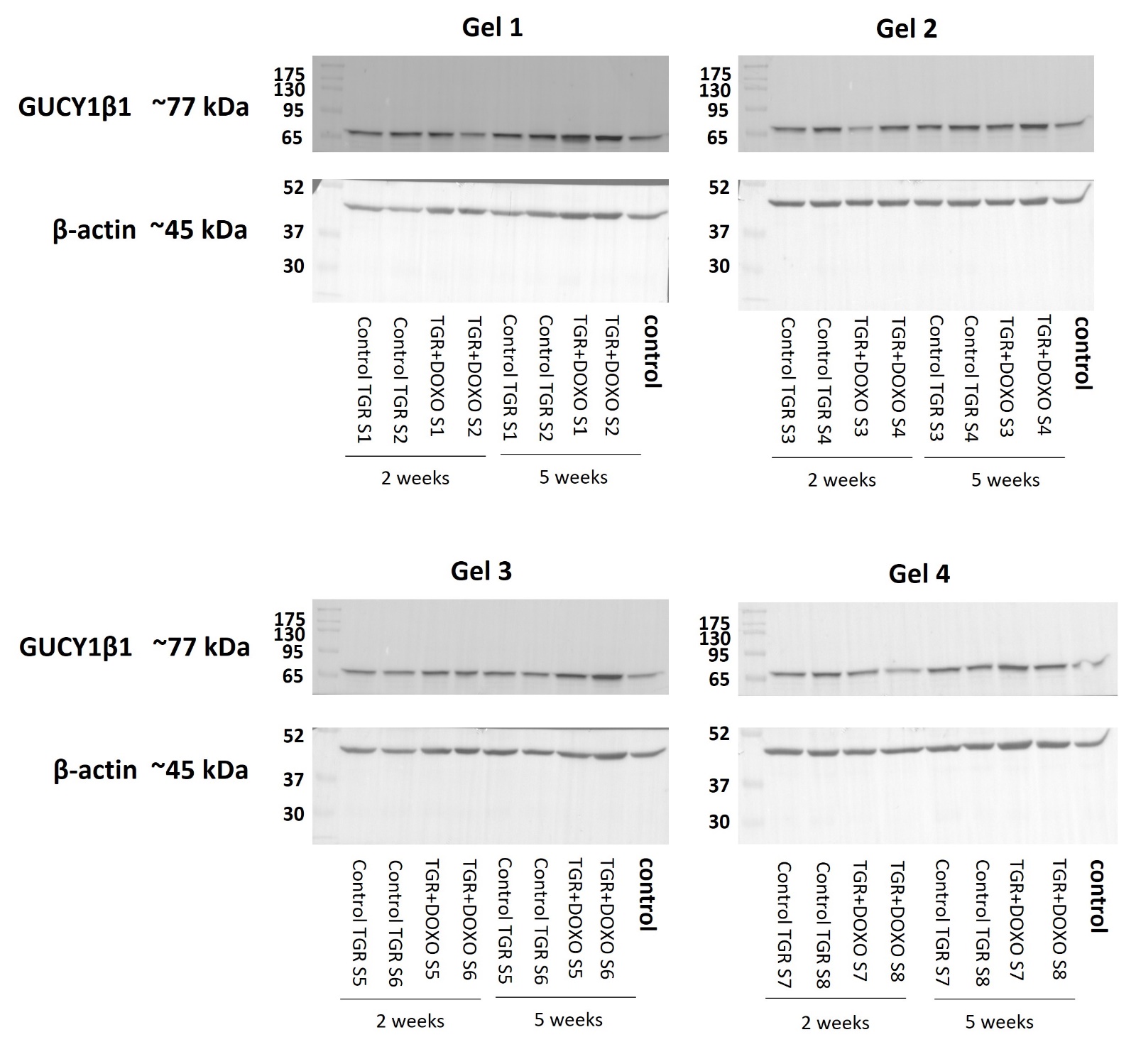


**FIGURE S6. Western blot (WB) images showing protein expression of sGC receptor (GUCY1β1) in
kidney** with housekeeping protein (β-actin) collected from Ren-2 transgenic hypertensive rats (TGR) treated with doxorubicin (DOXO) for five consecutive weeks (total dose 10 mg/kg of BW) after 2 and 5 weeks from the last DOXO injection in comparison to control TGR (placebo treated) group.


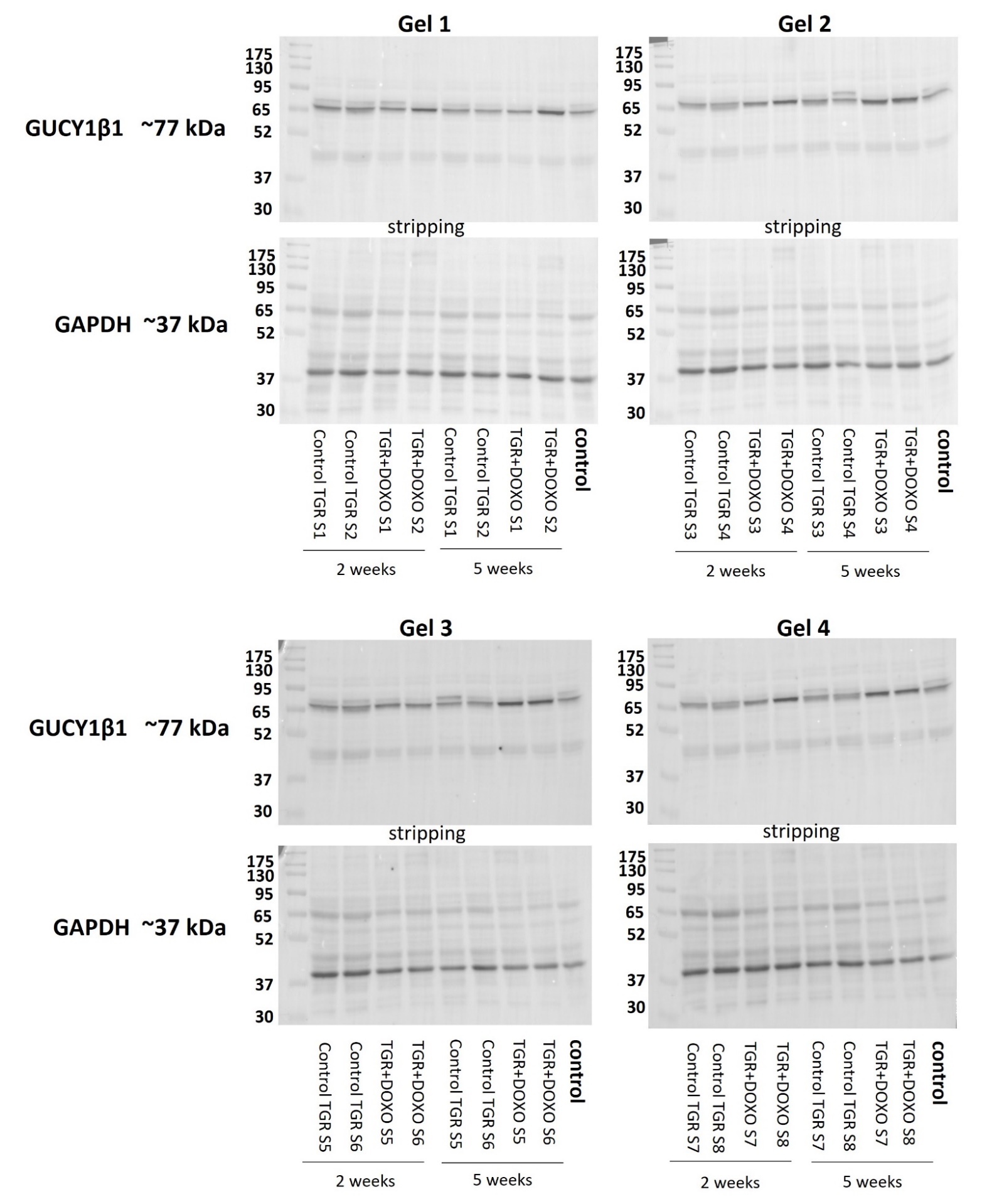


**FIGURE S7. Western blot (WB) images showing protein expression of sGC receptor (GUCY1β1) in
left ventricle** with housekeeping protein (GAPDH) collected from Ren-2 transgenic hypertensive rats (TGR) treated with doxorubicin (DOXO) for five consecutive weeks (total dose 10 mg/kg of BW) after 2 and 5 weeks from the last DOXO injection in comparison to control TGR (placebo treated) group.
